# Combined Inhibition of ATR and Ribonucleotide Reductase Induces Synergistic Antineoplastic Activity in Osteosarcoma Cells

**DOI:** 10.1002/cnr2.70568

**Published:** 2026-05-05

**Authors:** Natalie Aderhold, Lisa Immesberger, Sabine Becker, Till Milde, Bernd Gruhn, Jürgen Sonnemann

**Affiliations:** ^1^ Department of Paediatric and Adolescent Medicine Jena University Hospital, Friedrich Schiller University Jena Jena Germany; ^2^ Research Centre Lobeda Jena University Hospital, Friedrich Schiller University Jena Jena Germany; ^3^ Comprehensive Cancer Centre Central Germany (CCCG) Jena Germany; ^4^ Hopp Children's Cancer Center Heidelberg (KiTZ) Heidelberg Germany; ^5^ Clinical Cooperation Unit Pediatric Oncology German Cancer Research Center Heidelberg (DKFZ) Heidelberg Germany

**Keywords:** ATR, berzosertib, didox, osteosarcoma, ribonucleotide reductase, triapine

## Abstract

**Background:**

Osteosarcoma is the most common bone cancer in children and young adults. Its prognosis has not improved significantly since the introduction of the chemotherapy regimen established about 40 years ago, highlighting the need for new therapeutic strategies.

**Aims:**

The present study was undertaken to assess the effectiveness of combined inhibition of two promising drug targets, ATR and ribonucleotide reductase (RNR), in osteosarcoma cells.

**Methods and Results:**

The ATR inhibitor berzosertib and the RNR inhibitors triapine and didox were tested in *TP53* wild‐type (U2OS, MG‐63) and mutant (SaOS‐2) osteosarcoma cell lines. Combination effects were examined by flow cytometric analysis of cell death, loss of the mitochondrial membrane potential and DNA fragmentation as well as by caspase 3/7 activity assay and real‐time RT‐PCR. The drug interactions were evaluated using combination index analysis. Single treatment with ATR or RNR inhibitors resulted in mild to moderate effects, whereas combined treatment resulted in strong and synergistic effects. ATR and RNR inhibitors cooperated to elicit loss of the mitochondrial membrane potential, to activate caspase 3/7 and to trigger DNA fragmentation, suggesting that the combination of ATR and RNR inhibitors induced an apoptotic form of cell death. The cytotoxic effects were independent of *TP53* mutational status.

**Conclusion:**

Our study demonstrates that combined inhibition of ATR and RNR was effective in osteosarcoma cells. These in vitro findings offer support for investigating in vivo the potential of a combination of ATR and RNR inhibitors as a new treatment strategy for osteosarcoma.

## 1 Background

Osteosarcoma (OS) is the most common primary malignant bone tumour. It occurs most frequently in children and young adults between 10 and 30 years of age, with a first peak incidence at age 18 and a second at over 60 years of age [1, 2, 3]. The current standard of care treatment for OS was established in the 1980s and consists of induction chemotherapy, surgical resection and consolidation chemotherapy [4, 5]. In particular, the introduction of systemic chemotherapy markedly improved the prognosis: whereas the disease had previously been almost uniformly fatal, patients with localised disease now achieve a 5‐year event‐free survival rate of approximately 60% [1, 2]. However, survival rates have not improved during the last four decades [2], since the clinical efficacy of a combined regimen of methotrexate, doxorubicin and cisplatin was first demonstrated [6]. This is highly unsatisfactory, and it becomes even more unsatisfactory when considering survival rates of ~20% in patients with metastatic or relapsed disease [7]. The limited progress in improving OS outcomes stands in stark contrast to advances in other areas of paediatric oncology, particularly leukaemia [8], and underscores the urgent need for novel therapeutic strategies for OS.

Targeting DNA repair appears as a promising new approach [5]. *TP53* mutations are common in OS [9, 10], resulting in an impaired DNA damage response (DDR) [11]. This leads to a dependence on other DDR pathways, in particular the ATR/CHK1/WEE1 signalling cascade [12]. The latter is an important part of the DDR, as it is particularly crucial for the response to replication stress [13]. Cancer cells, especially those with mutant p53, exhibit higher replication stress levels than other cells [14] and thus rely on the ATR pathway to ensure their genomic stability under elevated replication stress. Therefore, components of the DDR such as ATR could be a vulnerability of OS cells and, as such, a drug target [3, 15, 16]. Recent studies have indeed demonstrated the effectiveness of ATR inhibitors (ATRi) in OS cells [17, 18].

Similar to other anticancer drugs, the development of drug resistance is also a common problem with targeted agents [19]. However, the emergence of drug resistance may be mitigated through the use of combination therapies [20]. In cancer cells, replication stress results from oncogene activation and the disruption of G1 checkpoint control [14]. Unconstrained replication requires coordinated replication fork progression and a sufficient quantity of deoxyribonucleotides (dNTPs) to maintain genomic stability under increased replication stress [21]. This points to ribonucleotide reductase (RNR) as a potential drug target, as it is the rate‐limiting enzyme in the de novo synthesis of dNTPs [22]. A recent study was the first to report the effectiveness of an RNR inhibitor (RNRi), namely, triapine (also known as 3‐AP), in OS cells [23].

These findings prompted us to evaluate whether the combination of ATRi and RNRi could surpass their antineoplastic effects as single agents. We found that ATRi combined with RNRi evoked OS cell death in a synergistic manner, indicating the therapeutic potential of ATRi–RNRi combination treatment in OS.

## 2 Materials and Methods

### 2.1 Cell Culture

U2OS (CVCL_0042) and SaOS‐2 (RRID: CVCL_0548) cells were purchased from the DSMZ (Braunschweig, Germany), and MG‐63 (CVCL_0426) cells were purchased from ATCC (Manassas, VA, USA). Cells were cultivated in RPMI 1640 medium (Capricorn Scientific, Ebsdorfergrund, Germany), supplemented with 10% foetal bovine serum (Capricorn Scientific), 100 units/mL penicillin G sodium and 100 units/mL streptomycin sulphate (Lonza, Basel, Switzerland). Cells were maintained in 75 cm^2^ cell culture flasks in a humidified incubator at 37°C and 5% CO_2_. Mycoplasma contamination was tested using the qPCR Mycoplasma Test Kit from Applichem (Darmstadt, Germany).

### 2.2 Treatment of Cells

U2OS cells were cultivated in 6‐well tissue culture plates, and MG‐63 and SaOS‐2 cells were cultivated in 12‐well tissue culture plates. For flow cytometric analysis, cells were seeded at a density of 110 000 cells per well, and for caspase 3/7 assay and PCR analysis, cells were seeded at a density of 200 000 cells per well. Cells were treated with the ATRi berzosertib (0.25–1 μM; Biozol Selleck Chemicals, Planegg, Germany) 24 h after seeding and with the RNRi triapine (0.2–1.6 μM; Biozol Selleck Chemicals) 1 h later, and cultured for either 24 h (caspase 3/7 assay and PCR) or 48 h (flow cytometric analysis). In the respective experiments, cells were pretreated with the pan‐caspase inhibitor z‐VAD‐fmk (20 μM; Enzo Life Sciences, Lörrach, Germany) 1 h before treatment with berzosertib.

### 2.3 Flow‐Cytometric Analysis of Cell Death, Mitochondrial Transmembrane Potential (Δ*ψ*_m_) Loss and DNA Fragmentation

Cell death was determined by propidium iodide (PI; Sigma‐Aldrich, Deisenhofen, Germany) uptake analysis. After harvesting, cells were incubated in 2 μg/mL PI in PBS for 5 min at 4°C in the dark. Δ*ψ*_m_ loss was determined by assessing 3,3′‐dihexyloxacarbocyanine iodide (DiOC_6_(3); Thermo Fisher Scientific, Dreieich, Germany) staining of mitochondria. Before harvesting, cells were incubated with 50 nM DiOC_6_(3) for 45 min at 37°C in the dark. DNA fragmentation was determined by assessing cells for PI incorporation into DNA. After harvesting, cells were washed twice with PBS and fixed in 70% ethanol at −20°C overnight. After washing, cells were resuspended in PBS containing 1% glucose, 2.5 μL/mL ribonuclease A (Roche, Mannheim, Germany) and 50 μg/mL PI and incubated at 4°C for 45 min in the dark. Ten thousand cells (cell death and Δ*ψ*_m_ loss) or 20 000 cells (DNA fragmentation) per sample were analysed on a BD FACS Canto II (Heidelberg, Germany) using BD FACSDiva software; events were gated to exclude debris and aggregates.

To examine the combination treatments for synergistic or antagonistic effects, the results of the cell death determinations were analysed by the combination index (CI) method according to Chou and Talalay [24] using Calcusyn software from Biosoft (Cambridge, UK). Theoretically, CI values of < 1, =1 and > 1 indicate synergism, additivism and antagonism, respectively. Here, only CI values < 0.9 were considered synergistic.

### 2.4 Caspase 3/7 Activity

Caspase 3/7 activity was determined by measuring the fluorescence of the caspase 3/7 substrate Ac‐DEVD‐AMC (Bachem, Weil am Rhein, Germany). After harvesting, cells were lysed in 10 mM Tris–HCl, 10 mM NaH_2_PO_4_/NaHPO_4_ (pH 7.5), 130 mM NaCl, 1% Triton X‐100 and 10 mM Na_4_P_2_O_7_ for 15 min at 4°C in the dark. Samples were mixed with 20 mM Hepes (pH 7.5), 10% glycerol, 2 mM DTT and 25 μg/mL Ac‐DEVD‐AMC. The release of AMC was measured at an excitation of 355 nm and an emission of 460 nm on a Tecan Infinite M200 Pro (Crailsheim, Germany) plate reader. Relative caspase 3/7 activities were calculated as the ratio of the emission of treated to untreated cells.

### 2.5 Real‐Time RT‐PCR

All procedures were performed according to the manufacturers' instructions. Briefly, total RNA was isolated using the Peqgold Total RNA Kit including DNase digestion (Peqlab, Erlangen, Germany), RNA was transcribed into cDNA using the Omniscript RT Kit (Qiagen, Hilden, Germany), and real‐time PCR was done on a Thermo Fisher Scientific Applied Biosystems 7900HT Real‐Time PCR system. Reactions were carried out in duplicate using Applied Biosystems Gene Expression Assays and Universal PCR Master Mix. The expression levels of *CDKN1A* (#Hs00355782_m1) and *BBC3* (#Hs00248075_m1) were normalised to *B2M* (#Hs00187842_m1) expression levels, and the relative gene expression levels were calculated by the 2(^–ΔΔ*C*^_t_) method.

### 2.6 Statistical Analysis

The results shown represent the mean ± SEM of each three independent experiments. A heteroscedastic, two‐tailed Student's *t*‐test was used for statistical analysis using Microsoft Excel (*p < 0.05, **p < 0.01, ***p < 0.001).

## 3 Results

### 3.1 ATRi and RNRi Synergise in Inducing Cell Death in OS Cells

As ATRi, we used berzosertib (also known as VE822, VX970 and M6620), a further development of the compound VE821 with improved pharmacological properties [25], which is being examined in phase I and II trials [26]. As RNRi, we used triapine and didox, drugs that have been tested in several phase I and II trials either as monotherapy or in combination with other anticancer compounds [27]. We tested the ATRi–RNRi combinations in three OS cell lines differing in their *TP53* status, namely U2OS, MG‐63 and SaOS‐2. U2OS and MG‐63 cells harbour wild‐type *TP53*, while SaOS‐2 cells are *TP53* deficient [28]. Regarding MG‐63 cells, however, it is important to note that they have shown very low levels of *TP53* mRNA and no detectable p53 protein [28, 29].

**FIGURE 1:**
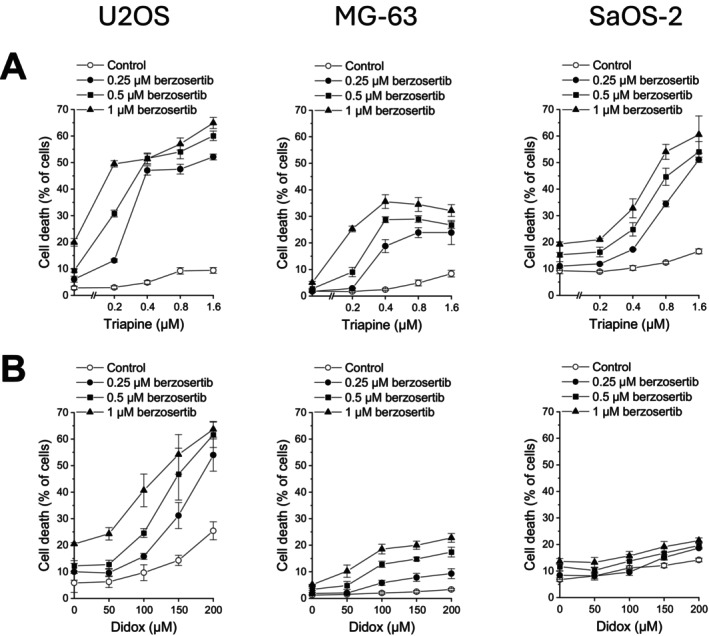
Berzosertib and RNRi cooperate in inducing cell death in OS cells. One hour after administration of berzosertib, cells were exposed to (A) triapine or (B) didox for another 48 h. Cell death was determined by flow‐cytometric analysis of PI uptake. Means ± SEM of each of the three independent measurements are shown.

As a first step, we determined berzosertib–triapine‐induced cell death by flow cytometric PI uptake analysis. One hour after treatment with berzosertib, cells were exposed to triapine for an additional 48 h. As shown in Figure 1A, OS cells were only marginally sensitive to triapine alone at the concentrations applied. Likewise, berzosertib as a single agent evoked only low (MG‐63) to moderate (U2OS, SaOS‐2) cell death. Yet when the two agents were combined, cell death amounted up to 64.9% ± 2.1% in U2OS cells, up to 35.6% ± 2.6% in MG‐63 cells, and up to 60.5% ± 7.0% in SaOS‐2 cells. We assessed the berzosertib–triapine combination for synergy using the CI method [24]. A synergistic interaction was observed for all combinations in U2OS cells (Table 1) as well for all combinations in MG‐63 cells except 0.25 μM berzosertib with 0.2 μM triapine (Table 2). In SaOS‐2 cells, a synergistic effect was seen for all combinations except 0.25 μM and 0.5 μM berzosertib with 0.2 μM triapine (Table 3).

**TABLE 1:** CI values for berzosertib plus triapine in U2OS cells.

Berzosertib (μM)	Triapine (μM)	CI
0.25	0.2	**0.478**
0.25	0.4	**0.073**
0.25	0.8	**0.082**
0.25	1.6	**0.081**
0.5	0.2	**0.272**
0.5	0.4	**0.112**
0.5	0.8	**0.108**
0.5	1.6	**0.091**
1.0	0.2	**0.231**
1.0	0.4	**0.218**
1.0	0.8	**0.178**
1.0	1.6	**0.131**

*Note:* Based on data from Figure 1a, CI values were calculated with the Chou–Talalay method. CI values in bold indicate a synergistic interaction.

**TABLE 2:** CI values for berzosertib plus triapine in MG‐63 cells.

Berzosertib (μM)	Triapine (μM)	CI
0.25	0.2	0.961
0.25	0.4	**0.118**
0.25	0.8	**0.137**
0.25	1.6	**0.247**
0.5	0.2	**0.333**
0.5	0.4	**0.080**
0.5	0.8	**0.119**
0.5	1.6	**0.228**
1.0	0.2	**0.122**
1.0	0.4	**0.081**
1.0	0.8	**0.115**
1.0	1.6	**0.198**

*Note:* Based on data from Figure 1a, CI values were calculated with the Chou–Talalay method. CI values in bold indicate a synergistic interaction (CI values > 0.9 were not considered synergistic).

**TABLE 3:** CI values for berzosertib plus triapine in SaOS‐2 cells.

Berzosertib (μM)	Triapine (μM)	CI
0.25	0.2	1.222
0.25	0.4	**0.529**
0.25	0.8	**0.073**
0.25	1.6	**0.018**
0.5	0.2	0.909
0.5	0.4	**0.310**
0.5	0.8	**0.047**
0.5	1.6	**0.021**
1.0	0.2	**0.873**
1.0	0.4	**0.247**
1.0	0.8	**0.039**
1.0	1.6	**0.023**

*Note:* Based on data from Figure 1a, CI values were calculated with the Chou–Talalay method. CI values in bold indicate a synergistic interaction (CI values > 0.9 were not considered synergistic).

To test for a potential class effect of RNRi in OS cells, we also investigated the structurally different RNRi didox. The combination of berzosertib with didox resulted in equivalent cell death in U2OS cells as the combination with triapine (Figure 1B). Accordingly, the CI analysis demonstrated synergy for all combinations except 0.25 and 0.5 μM berzosertib with 50 μM didox and 0.5 μM berzosertib with 100 μM didox (Table 4). In MG‐63 cells, didox as a single agent was nearly ineffective with a maximum of 3.3% ± 0.4% cell death, whereas in combination with berzosertib, didox‐elicited cell death reached 22.8% ± 1.6%. The CI analysis confirmed that all combinations produced a synergistic effect except the combination with the lowest concentrations of both agents (Table 5). In SaOS‐2 cells, the effects of berzosertib combined with didox were considerably weaker than those of berzosertib combined with triapine, and the CI analysis showed a synergistic effect only for the combinations with the two highest didox concentrations (Table 6).

**TABLE 4:** CI values for berzosertib plus didox in U2OS cells.

Berzosertib (μM)	Didox (μM)	CI
0.25	50	1.552
0.25	100	1.077
0.25	150	**0.563**
0.25	200	**0.286**
0.5	50	1.577
0.5	100	**0.730**
0.5	150	**0.320**
0.5	200	**0.224**
1.0	50	**0.841**
1.0	100	**0.397**
1.0	150	**0.272**
1.0	200	**0.224**

*Note:* Based on data from Figure 1b, CI values were calculated with the Chou–Talalay method. CI values in bold indicate a synergistic interaction (CI values > 0.9 were not considered synergistic).

**TABLE 5:** CI values for berzosertib plus didox in MG‐63 cells.

Berzosertib (μM)	Didox (μM)	CI
0.25	50	1.493
0.25	100	**0.378**
0.25	150	**0.279**
0.25	200	**0.245**
0.5	50	**0.683**
0.5	100	**0.175**
0.5	150	**0.152**
0.5	200	**0.125**
1.0	50	**0.421**
1.0	100	**0.175**
1.0	150	**0.161**
1.0	200	**0.133**

*Note:* Based on data from Figure 1b, CI values were calculated with the Chou–Talalay method. CI values in bold indicate a synergistic interaction.

**TABLE 6:** CI values for berzosertib plus didox in SaOS‐2 cells.

Berzosertib (μM)	Didox (μM)	CI
0.25	50	2.207
0.25	100	2.084
0.25	150	**0.805**
0.25	200	**0.551**
0.5	50	1.806
0.5	100	1.049
0.5	150	**0.746**
0.5	200	**0.560**
1.0	50	1.451
1.0	100	1.066
1.0	150	**0.693**
1.0	200	**0.558**

*Note:* Based on data from Figure 1b, CI values were calculated with the Chou–Talalay method. CI values in bold indicate a synergistic interaction (CI values > 0.9 were not considered synergistic).

### 3.2 Combination Treatment of Berzosertib With Triapine Elicits Apoptosis in OS Cells

**FIGURE 2:**
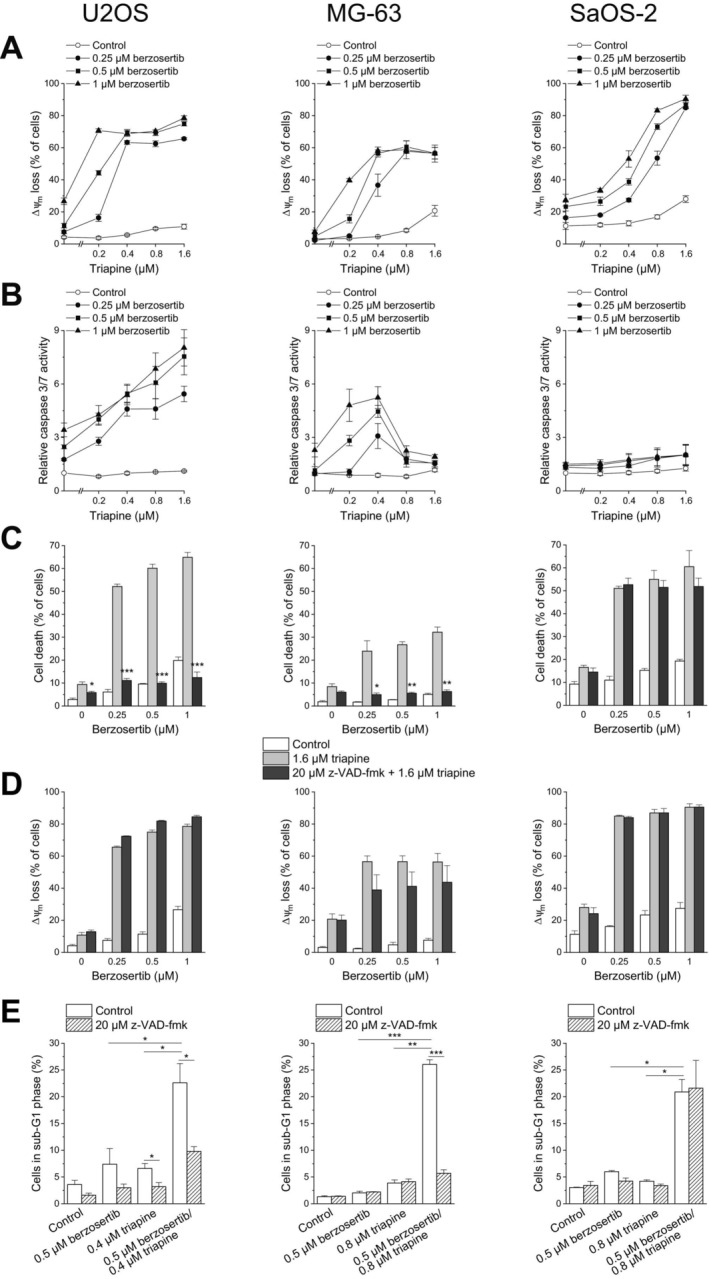
Berzosertib and triapine cooperate in inducing apoptosis in OS cells. One hour after administration of berzosertib, cells were exposed to triapine for another (B) 24 or (A, C–E) 48 h. (C–E) z‐VAD‐fmk was applied 1 h before treatment with berzosertib. (A, D) Loss of Δ*ψ*_m_ was determined by flow‐cytometric analysis of DiOC_6_(3) staining. (B) Caspase 3/7 activity was determined using the fluorogenic substrate Ac‐DEVD‐AMC; relative caspase 3/7 activities are the ratio of treated cells to untreated cells. (C) Cell death was determined by flow‐cytometric analysis of PI uptake. (E) Sub‐G1 cells were determined by flow‐cytometric analysis of PI‐stained ethanol‐fixed cells. Means ± SEM of each three independent measurements are shown (*p < 0.05, **p < 0.01, ***p < 0.001; (C, D) black bars vs. grey bars).

To gain insight into the nature of berzosertib–triapine‐induced cell death, we assessed the effects of the combination treatment using a range of readouts. Since most types of cell death, including apoptosis, engage mitochondria [30], we first examined the effect of the combination treatment by measuring Δ*ψ*_m_ loss using flow‐cytometric analysis of DiOC_6_(3) staining. Consistent with the results of the cell death determinations, berzosertib and triapine evoked weak to moderate effects as monotherapeutics, while their combination caused Δ*ψ*_m_ loss in up to 78.5% ± 1.4% of U2OS cells, up to 60.6% ± 3.7% of MG‐63 cells, and up to 90.4% ± 2.3% of SaOS‐2 cells (Figure 2A). As a second indicator of apoptotic cell death, we assessed the activation of caspases using the caspase 3/7 substrate Ac‐DEVD‐AMC after a 24‐h treatment. In U2OS and MG‐63 cells, the results of this assay corresponded to those of the cell death measurements. Berzosertib and triapine as single agents caused some activation of caspase 3/7, whereas their combination had a markedly enhanced effect (Figure 2B). In SaOS‐2 cells, however, the treatments led to only a slight increase in caspase 3/7 activity, despite marked Δ*ψ*_m_ loss.

To find out whether caspase activation was not only a bystander effect but crucial for berzosertib–triapine‐triggered cell death, we applied the pan‐caspase inhibitor z‐VAD‐fmk. As shown in Figure 2C, zC‐VAD‐fmk strongly reduced berzosertib–triapine‐induced cell death in U2OS and MG‐63 cells, yet had no effect in SaOS‐2 cells, thus in line with the weak caspase 3/7 activation in the latter. The pan‐caspase inhibitor did not impinge on berzosertib–triapine‐mediated Δ*ψ*_m_ loss in any of the cell lines, implying that caspases upstream of mitochondria were not involved (Figure 2D). To further corroborate the apoptosis‐inducing effect of berzosertib–triapine combination treatment, we determined DNA fragmentation by flow‐cytometric analysis of cells with DNA < 2*n* (sub‐G1 cells). This analysis demonstrates that berzosertib combined with triapine induced DNA fragmentation in all three cell lines (Figure 2E). It further demonstrates that z‐VAD‐fmk significantly diminished DNA fragmentation in U2OS and MG‐63 cells, but had no such effect in SaOS‐2 cells, again in line with the lack of substantial caspase 3/7 activation in these cells.

**FIGURE 3:**
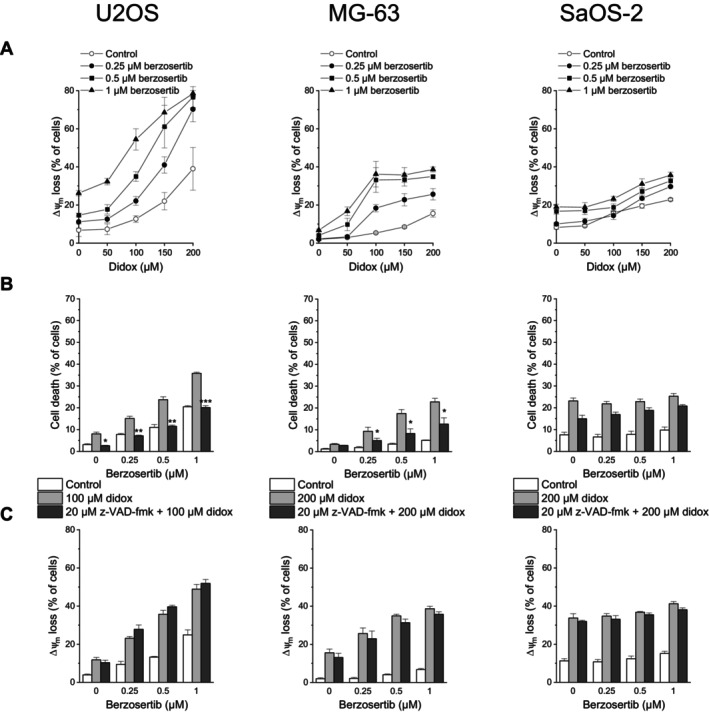
Berzosertib and didox cooperate in inducing apoptosis in OS cells. One hour after administration of berzosertib, cells were exposed to didox for another 48 h. (B, C) z‐VAD‐fmk was applied 1 h before treatment with berzosertib. (A, C) Loss of Δ*ψ*_m_ was determined by flow‐cytometric analysis of DiOC_6_(3) staining. (B) Cell death was determined by flow‐cytometric analysis of PI uptake. Means ± SEM of each three independent measurements are shown (*p < 0.05, **p < 0.01, ***p < 0.001; black bars vs. grey bars).

We investigated whether the combination of berzosertib with didox also mediated apoptotic cell death. As assessed by Δ*ψ*_m_ loss, the combination of these drugs resulted in a similar outcome as the combination of berzosertib with triapine in U2OS and MG‐63 cells (Figure 3A, compare Figure 2A). In SaOS‐2 cells, the berzosertib‐didox effect on Δ*ψ*_m_ loss was less pronounced, in keeping with the less pronounced cell death‐inducing activity of berzosertib‐didox combination treatment in these cells. z‐VAD‐fmk had a similar effect on cell death induced by berzosertib‐didox as on that induced by berzosertib–triapine: it decreased cell death in U2OS and MG‐63 cells, but hardly in SaOS‐2 cells (Figure 3B; compare Figure 2C). The pan‐caspase inhibitor again did not affect treatment‐elicited Δ*ψ*_m_ loss in any of the cell lines (Figure 3C; compare Figure 2D).

### 3.3 Combination Treatment of Berzosertib With Triapine Induces Gene Expression in OS Cells

**FIGURE 4:**
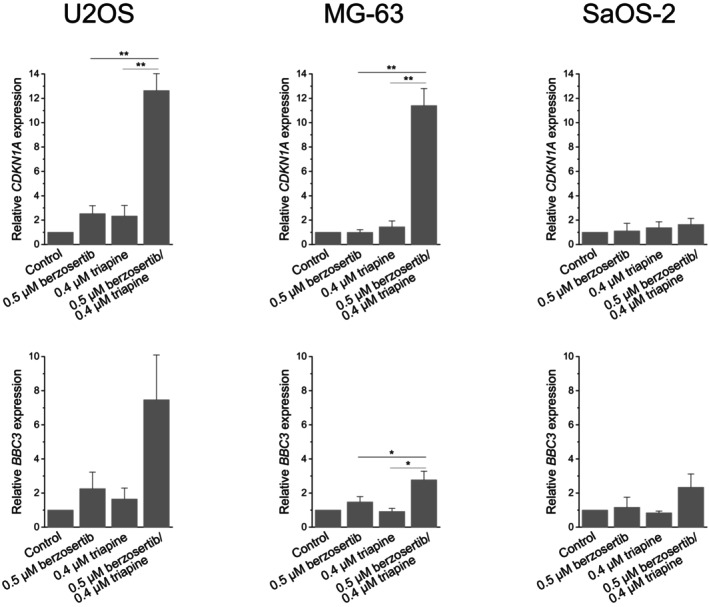
Berzosertib and triapine cooperate in inducing p53 target gene expression in OS cells. One hour after administration of berzosertib, cells were exposed to triapine for another 24 h. *CDKN1A* and *BBC3* expression levels were determined by real‐time RT‐PCR and normalised to *B2M* expression levels; relative gene expression levels are the ratio of treated cells to untreated cells. Means ± SEM of three independent measurements are shown (*p < 0.05, **p < 0.01).

We further questioned whether berzosertib combined with triapine had an impact on p53 target gene expression in OS cells. We focused on two important p53 target genes [31], *CDKN1A* (coding for the cyclin‐dependent kinase inhibitory protein p21) and *BBC3* (coding for the proapoptotic BCL‐2 family protein PUMA). The real‐time RT‐PCR determinations revealed that berzosertib–triapine combination treatment greatly increased gene expression in U2OS and MG‐63 cells, while berzosertib or triapine single‐agent treatment produced only a minor effect (Figure 4). Although MG‐63 cells do not have detectable p53 protein expression [28], residual p53 activity may be sufficient to drive the expression of target genes. The treatments had virtually no effect on *CDKN1A* and *BBC3* gene expression in the p53‐deficient SaOS‐2 cells.

## 4 Discussion

The present study explores the therapeutic value of combined inhibition of ATR and RNR in OS cells. It shows that the ATRi berzosertib cooperated synergistically with the RNRi triapine and didox in exerting antineoplastic effects in OS cells. ATRi and RNRi have been tested in a few studies for their effectiveness as single agents in OS [17, 18, 23], yet for all we know, our study is the first to investigate the combined targeting of ATR and RNR in OS.

Most importantly, our study reveals that the combination of berzosertib with triapine or didox considerably surpassed the drugs' individual effects by interacting in a synergistic manner at the majority of concentrations tested. The present study thus both complements and extends our recent study on the combination of ATRi with RNRi in another childhood tumour, Ewing's sarcoma (ES) [32], the second most common primary bone cancer in children and young adults [33]. Taken together, these studies demonstrate that ATRi–RNRi combination treatments were similarly effective in OS and ES cells. This is a remarkable finding since these two sarcoma types are genetically very distinct: OS has one of the highest mutational rates among paediatric cancers, ES one of the lowest [34, 35]. While OS is characterised by chromothripsis [10, 34] resulting in multiple genomic alterations [5, 36], ES has a very low mutational burden [37, 38] but is defined by a pathognomonic *FET::ETS* gene fusion, most commonly *EWSR1::FLI1*, which encodes the fusion protein EWS::FLI1 [33]. Hence, ES has a natural target, the oncoprotein EWS::FLI1, whereas OS has no such distinct target due to its genomic heterogeneity. However, OS and ES cells have one thing in common: they both suffer from elevated replication stress [39, 40], although the levels and underlying mechanisms differ, and thus rely on DDR pathways to survive. Both OS and ES share features with BRCA1/2‐mutant cancers [41, 42, 43], referred to as ‘BRCAness’ [44], rendering them sensitive to treatments that exploit DNA repair deficiencies. Targeting DDR‐related vulnerabilities therefore appears as a promising therapeutic avenue for both tumours [21, 45].

Here and in our previous study [32], we have demonstrated that the combination of ATRi and RNRi was effective in OS and ES cells, respectively, suggesting that this combination regimen may be a viable treatment option for both tumours. In ES, the utility of ATR pathway inhibitors combined with RNRi is also supported by studies on the cooperative activity of RNRi in combination with inhibitors of the ATR downstream effector kinases CHK1 and WEE1 [46, 47, 48, 49], but none of these combinations have yet been addressed in OS. The mechanism underlying the beneficial interaction of combined ATR and RNR inhibition is so far not fully understood. Yet a plausible hypothesis is that RNR inhibition diminishes the concentration of dNTPs, thereby escalating the already high replication stress in cancer cells. This leads to the activation of ATR that serves to manage replication stress. ATRi neutralise this protective response, ultimately resulting in cancer cell death [50]. Another hypothesis is that ATR inhibition leads to the degradation of the RNR subunit RRM2 [51] and thus to an even more critical reduction of dNTPs. The latter consideration is consistent with the finding that ATR fosters the stabilisation of RRM2 by downregulating the RRM2‐degrading SCF (cyclin F) ubiquitin ligase complex [52]. Our study does not allow for a distinction between these hypotheses, as it was not designed to provide an in‐depth mechanistic explanation.

Our results suggest that the mechanisms underlying cell death induced by ATRi–RNRi combination treatment varied between the cell lines explored. In p53 wild‐type U2OS and MG‐63 cells, it triggered the mitochondrial pathway of apoptosis, as determined by evaluating a number of features characteristic of apoptosis. ATRi–RNRi combination treatment resulted in Δ*ψ*_m_ loss, indicating the induction of the mitochondrial pathway of apoptosis, as well as caspase 3/7 activation and sub‐G1 cell accumulation. The use of the pan‐caspase inhibitor z‐VAD‐fmk further confirmed the induction of apoptosis, as it prevented cell death and DNA fragmentation, thus pointing to a predominantly caspase‐dependent cell death mechanism induced by ATRi–RNRi in U2OS and MG‐63 cells. In contrast, in p53‐null SaOS‐2 cells, ATRi–RNRi barely induced caspase 3/7 activation, and consistently, z‐VAD‐fmk did not reduce cell death and DNA fragmentation. This indicates that the combination of ATRi and RNRi treatment can mediate either caspase‐dependent or ‐independent cell killing in OS cells, a finding likely related to the high intertumoural heterogeneity in OS [7]. It more specifically indicates that ATRi–RNRi‐induced caspase activation may rely on p53, in keeping with the effects of adenoviral expression of wild‐type p53 in SaOS‐2 cells; in this study, caspase activation was only observed in p53‐expressing cells, but not in control ones [53]. In any case, the important result from a clinical point of view is that the ATRi–RNRi combination was comparably effective in p53‐proficient and ‐deficient OS cells. Consistent with these findings, another study demonstrated that p53 does not play a role in apoptosis induced by severe DNA damage, impaired protein turnover, or spindle misassembly in OS cells [54]. It should be noted, however, that the apoptotic responses in our study differed between p53 wild‐type and p53‐null cells. Therefore, it cannot be ruled out that the clinical efficacy of the combination therapy depends on the p53 mutational status in a way that cannot be investigated in an in vitro study.

This study has some limitations. It aimed at exploring in vitro whether ATRi in combination with RNRi could exert synergistic anticancer effects on OS cells. We thus cannot make any statement about the in vivo effectiveness and possible side effects of the combined ATRi–RNRi treatment. Future xenograft studies could answer these questions. We also cannot make any statement about the clinical feasibility of the drug combination and its therapeutic window. However, we restricted our study to drugs that have already been examined in clinical trials to facilitate the potential clinical translation of the results and abstained from any genetic experiments.

ATRi and RNRi both have potential as anticancer drugs. The findings presented here demonstrate that their effectiveness could be further improved by their combination. Our in vitro study therefore points to the ATRi–RNRi combination as a new treatment option for patients with OS and provides a rationale for investigating this combination approach in vivo.

## Author Contributions

**Natalie Aderhold:** writing – original draft, conceptualization, investigation, methodology, validation, visualization, software, formal analysis, data curation, writing – review and editing. **Lisa Immesberger:** investigation, writing – review and editing, methodology, data curation. **Sabine Becker:** investigation, methodology, writing – review and editing, data curation. **Till Milde:** writing – review and editing, conceptualization. **Bernd Gruhn:** conceptualization, writing – review and editing, supervision, project administration. **Jürgen Sonnemann:** conceptualization, supervision, writing – review and editing, project administration, investigation, methodology, validation, visualization, data curation.

## Funding

The authors have nothing to report.

## Ethics Statement

The authors have nothing to report.

## Consent

The authors have nothing to report.

## Conflicts of Interest

The authors declare no conflicts of interest.

## Data Availability

The data that support the findings of this study are available from the corresponding author upon reasonable request.
